# Examining the impact of dimethyl sulfide emissions on atmospheric sulfate over the continental U.S.

**DOI:** 10.3390/atmos14040660

**Published:** 2023-03-31

**Authors:** Golam Sarwar, Daiwen Kang, Barron H. Henderson, Christian Hogrefe, Wyat Appel, Rohit Mathur

**Affiliations:** 1 Center for Environmental Measurement and Modeling, Office of Research and Development, U.S. Environmental Protection Agency, Research Triangle Park, North Carolina, USA; 2 Office of Air Quality Planning and Standards, U.S. Environmental Protection Agency, Research Triangle Park, NC 27711, USA

**Keywords:** DMS, seawater, SO_2_, sulfate, CMAQ

## Abstract

We examine the impact of dimethylsulfide (DMS) emissions on sulfate concentrations over the continental U.S. by using the Community Multiscale Air Quality (CMAQ) model version 5.4 and performing annual simulations without and with DMS emissions for 2018. DMS emissions enhance sulfate not only over seawater but also over land, although to a lesser extent. On an annual basis, the inclusion of DMS emissions increase sulfate concentrations by 36% over seawater and 9% over land. The largest impacts over land occur in California, Oregon, Washington, and Florida, where the annual mean sulfate concentrations increase by ~25%. The increase in sulfate causes a decrease in nitrate concentration due to limited ammonia concentration especially over seawater and an increase in ammonium concentration with a net effect of increased inorganic particles. The largest sulfate enhancement occurs near the surface (over seawater) and the enhancement decreases with altitude, diminishing to 10–20% at an altitude of ~5 km. Seasonally, the largest enhancement of sulfate over seawater occurs in summer, and the lowest in winter. In contrast, the largest enhancements over land occur in spring and fall due to higher wind speeds that can transport more sulfate from seawater into land.

## 1. Introduction

Sulfate is an important component of atmospheric fine particles and can also affect the cloud condensation nuclei and the aerosol radiative forcing [1–4]. Sulfate is formed in the atmosphere mainly from the oxidation of sulfur dioxide (SO_2_) via gas- and aqueous-phase chemistry. Sulfur can be emitted from natural as well as anthropogenic sources. Significant reductions of anthropogenic SO_2_ emissions from fossil fuel combustion have occurred in the U.S. during the past decades due to stringent control measures in response to tightened air quality standards. For example, the U.S. Environmental Protection Agency (EPA) reported a 93% reduction of anthropogenic SO_2_ emissions during 1980–2021 despite a 187% increase in gross domestic product, 111% increase in vehicle miles travelled, and 25% increase in energy consumption during the same period (https://www.epa.gov/air-trends/air-quality-national-summary#air-quality-trends (accessed on 24 August 2022)). Similar changes have occurred in many other parts of the world [5]. The contribution of sulfate to fine particles varies with time and location. Tanner et al. [6] analyzed trends in annual pollutant concentrations in eastern Tennessee and reported that ammonium sulfate contributed 48% in 1999 and 41% in 2013 to fine particle mass concentrations at Look Rock, Tennessee. Malm et al. [7] performed analysis of measured sulfate concentrations at the IMPROVE (Interagency Monitoring of Protected Visual Environments) sites for 2001–2015 and reported that, on average, ammonium sulfate contributed 30% to the summertime reconstructed fine particle mass. There are some specific regions in the U.S. where other species contributes a substantial fraction to fine particle mass. For example, aerosol nitrate and organic aerosol contribute ~85% of wintertime fine particle mass in San Joaquin Valley of California [8]. Atmospheric sulfate concentrations in the U.S. have decreased over the years; however, it is still an important constituent of fine particles.

Dimethyl sulfide (DMS; CH_3_SCH_3_) can be emitted from natural as well as anthropogenic sources. Natural sources of DMS include emissions from ocean, wetland, and plant and soil with ocean being the largest source [9]. Phytoplankton in seawater [9] contain dimethylsulfoniopropionate (DMSP) which produces DMS upon breakdown. DMS emissions can also be emitted from anthropogenic sources. For example, paper and pulp industries and rayon/cellulosics manufacturing facilities can emit DMS; however, their emissions are an order of magnitude lower than natural emissions [10]. Estimates of oceanic DMS emissions range between 17.6–34.4 Tg S yr^−1^ [3] and are ~10 times larger than anthropogenic DMS emission estimates of 2.20 Tg S yr^−1^ [10]. Once emitted into the atmosphere, oxidation of DMS forms sulfur-containing products such as SO_2_ and sulfuric acid [11–12]. These products then lead to the formation of sulfate aerosols. While the anthropogenic sulfur emissions are decreasing, natural sulfur emissions remain mostly unchanged. Thus, natural oceanic DMS emissions play an increasingly important role in the atmosphere, and their impact on sulfate needs a careful examination. Several previous studies have been conducted to examine the impact of natural sulfur emissions on sulfate [3, 13–20]. However, most of these studies employed global or hemispheric models and were conducted with older modeling systems for historic conditions not reflecting recent decreases in anthropogenic sulfur emissions, and/or covered only limited areas over the U.S. For example, Park and co-workers [13] used the GEOS-Chem version 5.03 model (geos-chem.seas.harvard.edu) with a horizontal resolution of 2° × 2.5° and conducted simulations for 2001. They included natural sulfur emissions from oceans, volcanoes, and biomass burning sources and reported that these emissions can enhance ammonium sulfate concentrations in the U.S. Zhao et al. [20] implemented oceanic DMS emissions in the CMAQv5.3 modeling system [21]. Using the hemispheric Community Multiscale Air Quality (CMAQ) model (www.epa.gov/cmaq) version 5.3 with 108-km horizontal grids, they reported that DMS emissions can enhance surface sulfate concentration over many areas of the Northern Hemisphere including the U.S., but did not conduct any regional-scale CMAQ simulations. In one of the two previous regional-scale modeling studies we are aware of, Mueller et al. [18] applied CMAQv4.6 with a horizontal resolution of 36 × 36 km for 2002 atmospheric conditions to examine the impact of natural emissions on sulfate. They included natural sulfur emissions from ocean, coastal wetlands, fresh water lakes, the Great Salt Lakes, soil, and geothermal sources, performed simulations over the continental U.S., Canada, Mexico, and surrounding oceanic areas, and reported that these emissions can enhance ammonium sulfate over the U.S. Perraud et al. [19] examined the impacts of a hypothetical scenario with zero anthropogenic SO_2_ emissions in a large coastal urban area in California. They applied a three-dimensional air quality model (The University of California, Irvine – California Institute of Technology regional airshed model) with 5-km horizontal grids over California and reported that particle formation will be reduced by two orders of magnitude in the case of zero anthropogenic SO_2_ emissions; however, particles will continue to form from natural and anthropogenic sources of organosulfur compounds. In this study, we add to this body of knowledge generated in earlier studies by examining the impact of oceanic DMS emissions on sulfate over the continental U.S. for recent atmospheric conditions using the latest version of CMAQ (version 5.4) with horizontal grid-spacings of 12-km and constrained by lateral boundary conditions from corresponding large-scale CMAQv5.4 simulations performed over the northern hemisphere.

## 2. Materials and Methods

To quantify the impact of oceanic DMS, we use a simulation without DMS emissions (NODMS) and a simulation with DMS emission (WDMS). They both share the base model configuration and emissions (except DMS). The WDMS simulation uses the on-line implementation of DMS emissions while the NODMS simulation does not use the on-line DMS emissions. Both simulations include additional chemistry implemented for DMS. The base model configuration, the additional chemistry, and DMS emissions implementations are described below.

### 2.1 Base Model Configuration

CMAQ is a state-of-the-science three-dimensional chemical transport model containing detailed treatments of important atmospheric processes. It has been widely used in the U.S. and many other countries for air quality simulations involving research as well as regulatory activities [21–25]. Here, we use the recently released CMAQv5.4 [26] for simulating air quality for the entire year of 2018. The modeling domain covers the entire continental U.S., parts of Canada and Mexico, and surrounding oceanic areas with 12-km horizontal grid spacings and 35 vertical layers of varying thickness with a surface layer height of 20 meters. The modeling domain contains a total of 459 × 299 × 35 (4,803,435) grid-cells. Meteorological fields were generated using the Weather Research and Forecasting (WRFv4.3.3, [27]) model with the RRTMG (Rapid Radiative Transfer Model for GCMs) for long-and short-wave radiation [28], Kain-Fritsch scheme [29] with lightning assimilation [30–31] for convective parameterization, Morrison microphysics scheme [32], Pleim-Xiu land surface model [33–34] and the Asymmetric Convective Model version 2 (ACM2; [35–36]) for planetary boundary layer (PBL) scheme. Four-dimensional data assimilation (FDDA) using “analysis nudging” was applied to continuously nudge wind, temperature and water vapor mixing ratio above the PBL [37] toward the NAM 12-km analysis every three hours. The Meteorology Chemistry Interface Processor (MCIPv5.3.3) [38] was used to process the WRF results into CMAQ-ready input files. Torres-Vazquez et al. [39] compared WRFv4.1.1 predictions with observed data over the U.S. (same modeling domain used in this study) and reported morel performance for wind speed, wind direction, temperature, and mixing ratios. The model configuration between the two studies is same except that we use WRFv4.3.3 and they used WRFv4.1.1. The WRF model performance in this study is similar to that reported by Torres-Vazquez et al. [39]. We employ the standard cloud and aero7 modules, and the Carbon Bond chemical mechanism (version 6, release 5 (CB6r5)) [40] with chlorine chemistry [41].

The model includes emissions from various natural and anthropogenic sources. Anthropogenic source emissions include contributions from mobile, point, and non-point sources and are obtained from the 2017 National Emissions Inventory and projected for 2018. Additional information on different types of emissions can be found in EPA [42]. Mobile source emissions include contributions from on- and off-road vehicles, commercial marine vessels, and railroads. Point source emissions include contributions from electric generating units, oil and gas facilities, industrial sources not included in electric generating units and oil/gas facilities, agricultural field burning activities, wild/prescribed burning activities, and airports. Non-point source emissions include contributions from agriculture, area fugitive dust, oil and gas facilities, residential wood combustion and others. In addition, emissions from Mexico and Canada are also included. The details of these emissions can be found at the EPA website (www.epa.gov/cmaq/equates#emissions_modeling). Biogenic emissions are calculated using the in-line Biogenic Emission Inventory System (BEIS4). In-line sea-salt spray emissions are calculated following Gantt et al. [43] while in-line lightning emissions are calculated following Kang et al. [44]. The simulation in this study does not include any wind-blown dust emissions.

### 2.2 DMS Emission Implementation

In addition, we include DMS flux (F) from the ocean estimated using the total gas transfer velocity (${\text{k}}_{\text{T}}$) and the DMS concentration in seawater (${\text{C}}_{\text{w}}$) (F) as follows [3]:

(1) $$\text{F}\;=\;{\text{k}}_{\text{T}}\times {\text{C}}_{\text{w}}$$

DMS concentrations in seawater can be obtained from several databases [3, 45–46]. Here, we use the monthly climatological seawater concentration from the Surface Ocean and Lower Atmosphere project (www.bodc.ac.uk/solas_integration/implementation_products/group1/dms) [3] for calculating DMS flux. The ${\text{k}}_{\text{T}}$ in equation (1) is calculated using the water side gas transfer velocity (${\text{k}}_{\text{w}}$) and the atmospheric gradient fraction (γ) as follows:

(2) $${\text{k}}_{\text{T}}\;=\;{\text{k}}_{\text{w}}\times \;(1-\text{γ})$$

${\text{k}}_{\text{w}}$ can be estimated using several parameterizations [47–49]. Lana et al. [3] estimated global DMS emissions using these parameterizations and reported that the Wanninkhof [49] parameterization produces the highest estimates and the Liss and Merlivat [47] parameterization produces the minimum estimates. Consistent with the study of Zhao et al. ([20] for the Northern Hemisphere, we also use the Liss and Merlivat [47] parameterization (equations (3)) to calculate DMS flux.


(3) $$k_{w}=\left\{ \begin{array}{l} 0.17\times {\text{U}}_{10}/{\left(S_{c\text{DMS}\;}/600\right)}^{2/3}\;\;{\text{U}}_{10}\leq 3.6\;\text{m}/\text{s}\\ \left(2.85\times {\text{U}}_{10}-9.65\right)/{\left(S_{c\text{DMS}\;}/600\right)}^{1/2}\;\;3.6<{\text{U}}_{10}\leq 13\;\text{m}/\text{s}\\ \left(5.9\times {\text{U}}_{10}-49.3\right)/{\left({\text{S}}_{c\text{DMS}}/600\right)}^{1/2}\;\;{\text{U}}_{10}>13\;\text{m}/\text{s} \end{array}\right.$$

Schmidt number (${Sc}_{DMS}$) describes the diffusion of DMS in seawater and is calculated using sea surface temperature $\left({\text{T}}_{\text{Sw}},{\;}^{\circ }\text{C}\right)$ [50]:

(4) $${Sc}_{DMS}=2674.0-147.12\times T_{Sw}+3.726\times {T_{Sw}}^{2}-0.038\times {T_{Sw}}^{3}$$

The atmospheric gradient fraction, γ, can be calculated using equation 5:

(5) $$\text{γ}=1.0/(1.0+{\text{k}}_{\text{a}}\text{/H}{\text{k}}_{\text{w}})$$

H is dimensionless Henry’s Law Coefficient (liquid/gas) and can be calculated as follows:

(6) $$\text{H}\;=\;{\text{h}}_{\text{Dacey}}\;\text{×}\;\text{R}\;\text{×}\;{\text{T}}_{\text{Sw}}$$

where R is the universal gas constant (0.08205746 atm L/K mol), ${\text{T}}_{\text{Sw}}$ is the sea surface temperature, and ${\text{h}}_{\text{Dacey}}$ is the solubility of DMS:

(7) $${\text{h}}_{\text{Dacey}}\;=\;\frac{1.0}{e^{\left(-\frac{3547.0}{273.15\;+\;T_{SW}\;}\right)\;+12.64}}$$

The wind speed-dependent airside transfer coefficient, ${\text{k}}_{\text{a}}$ is defined by

(8) $${\text{k}}_{\text{a}\;}=\;659\;\times {\;\text{U}}_{\text{10}}\sqrt{\left(\frac{M_{DMS}}{M_{H_{2}O}}\right)}$$

where ${\text{M}}_{\text{DMS}}$ is the molecular weight of DMS and ${\text{M}}_{{\text{H}}_{\text{2}}\text{O}}$ is the molecular weight of water [51].

**Figure 1.**
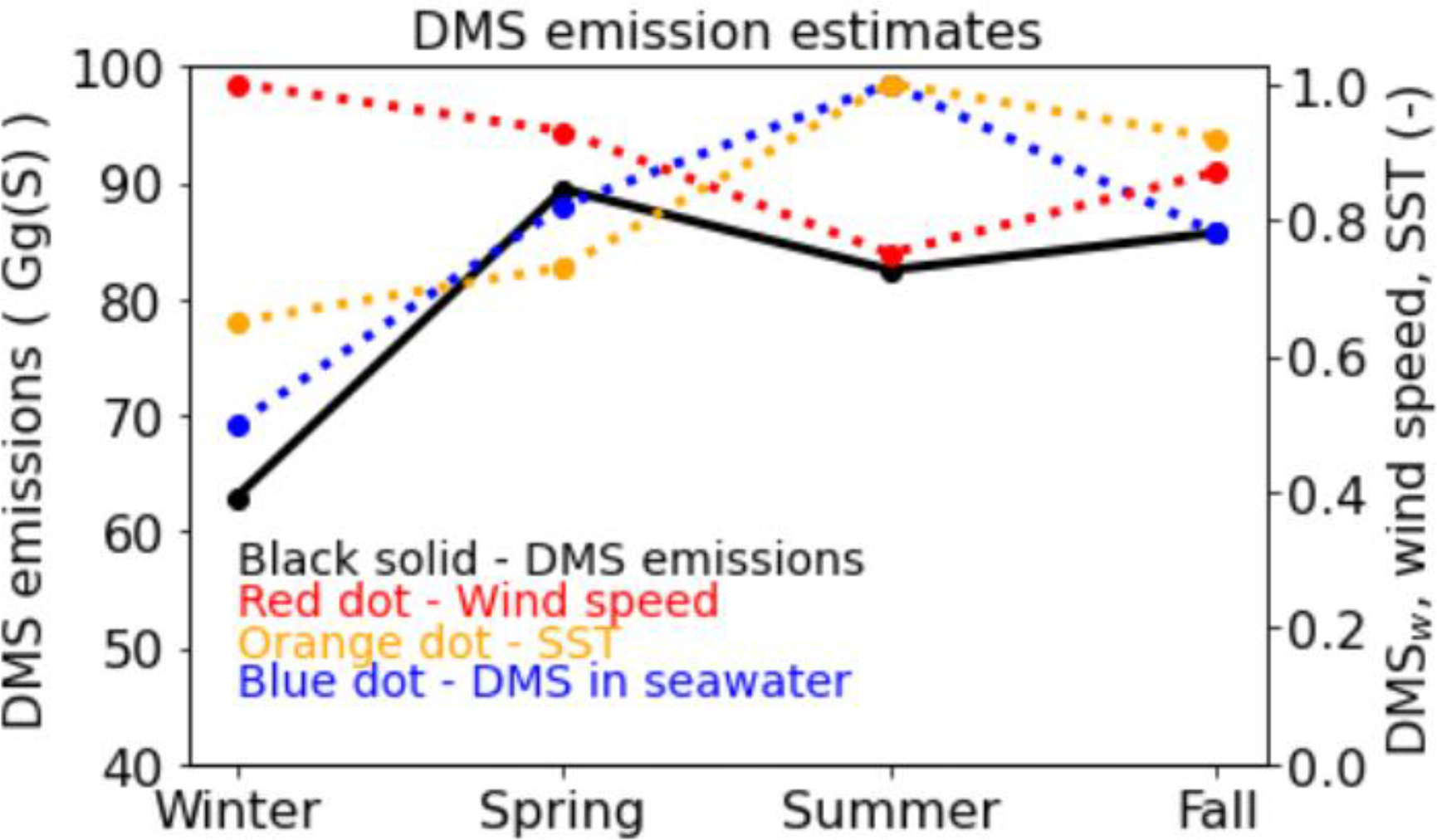
Seasonal DMS emissions over the modeling domain calculated using the Liss and Merlivat [34] parameterization for the water side gas transfer velocity (${\text{k}}_{\text{w}}$). DMS_w_, wind speed, and SST values shown on the right y-axis are dimensionless. Normalized values are calculated by dividing the actual values by their maximum values. Winter represents December-February, spring represents March-May, summer represents June-August, and fall represents September-November.

Seasonal DMS emissions (estimated using the scheme described above) over the modeling domain (only grid-cells emitting DMS emissions are used for the calculation) are shown in Figure 1. The highest estimate (89.5 Gg(S) - gigagram of sulfur) occurs in spring (March-May) and the lowest (62.9 Gg(S)) in winter. DMS emissions estimates in summer (June-August) and fall (September-November) are lower than those in spring but higher than those in winter (December-February). Mean DMS concentration in seawater, wind speed, and sea surface temperature (SST) are also shown in Figure 1. DMS emissions increase with higher DMS concentration in seawater, higher wind speed, and higher SST. Both the DMS concentration in seawater and SST are the highest in summer and the lowest in winter. However, wind speed is the highest in winter and the lowest in summer. Summertime DMS emissions are lower than those in spring due to lower wind speed. The lowest DMS emissions in winter are driven by the lowest DMS concentration in seawater and SST. The annual DMS emissions are 320 Gg(S) with ~21 Gg(S) in January and ~29 Gg(S) in July. Smith and Mueller [52] used a similar domain extent and reported 18 Gg(S) in January and 45 Gg(S) in July. January emissions estimates between the two studies vary by <20% while July emissions estimates vary by ~50% due likely to the differences in temporal allocation of annual emissions between the two studies.

### 2.3 DMS Chemistry Implementation

DMS chemistry was previously implemented in the hemispheric CMAQ model [20]. Seven chemical reactions involving oxidation of DMS by hydroxyl radical (OH), nitrate radical (NO_3_), chlorine radical (Cl), chlorine oxide (ClO), bromine oxide (BrO), and iodine oxide (IO) were used in the hemispheric CMAQ model. The CB6r5 chemical mechanism does not include any bromine or iodine chemistry; thus, the reactions of DMS with BrO and IO are not included in this study. In addition, since the reaction of ClO only contributes ~0.1% of the total DMS oxidation [20]; it is also excluded in this study. As shown in Table 1, four chemical reactions for DMS oxidation are included. DMS chemistry produces SO_2_ which is then further oxidized by gas-phase reaction with OH to produce sulfate aerosol. The in-cloud oxidation of SO_2_ by hydrogen peroxide (H_2_O_2_), ozone (O_3_), methyl hydroperoxide (MEPX), peroxyacetic acid (PACD), and oxygen catalyzed by iron (Fe[III]) and manganese (Mn[II]) further produces aerosol sulfate.

### 2.4 Boundary Conditions

Boundary conditions were generated from the hemispheric CMAQ model [56] simulations using CMAQv5.4. Two different model simulations were performed using the hemispheric CMAQv5.4: one simulation without DMS emissions and the other with DMS emissions. Results obtained with these hemispheric simulations were used to prepare two different sets of boundary conditions for the 12 km modeling domain for this study. The first 12 km simulation contained no DMS emissions and used the hourly boundary condition derived from the hemispheric model without DMS emissions (NODMS); the second 12 km simulation contained DMS emissions and used the hourly boundary condition derived from the hemispheric model with DMS emissions (WDMS). The differences in the model results are thus solely attributable to the DMS emissions that occur in both the 12 km and hemispheric domain. Initial conditions for the model were prepared using results from a previously generated model simulation for the EPA’s Air QUAlity TimE Series (EQUATES) (www.epa.gov/cmaq/equates). The simulation was started on December 22, 2017 and continued until December 31, 2018. The simulations for the first 10 days were used for spin-up purpose. The analyses of the model results were performed for the entire year of 2018.

## 3. Results and discussion

### 3.1 Annual impact of DMS emissions on surface inorganic particles

**Figure 2.**
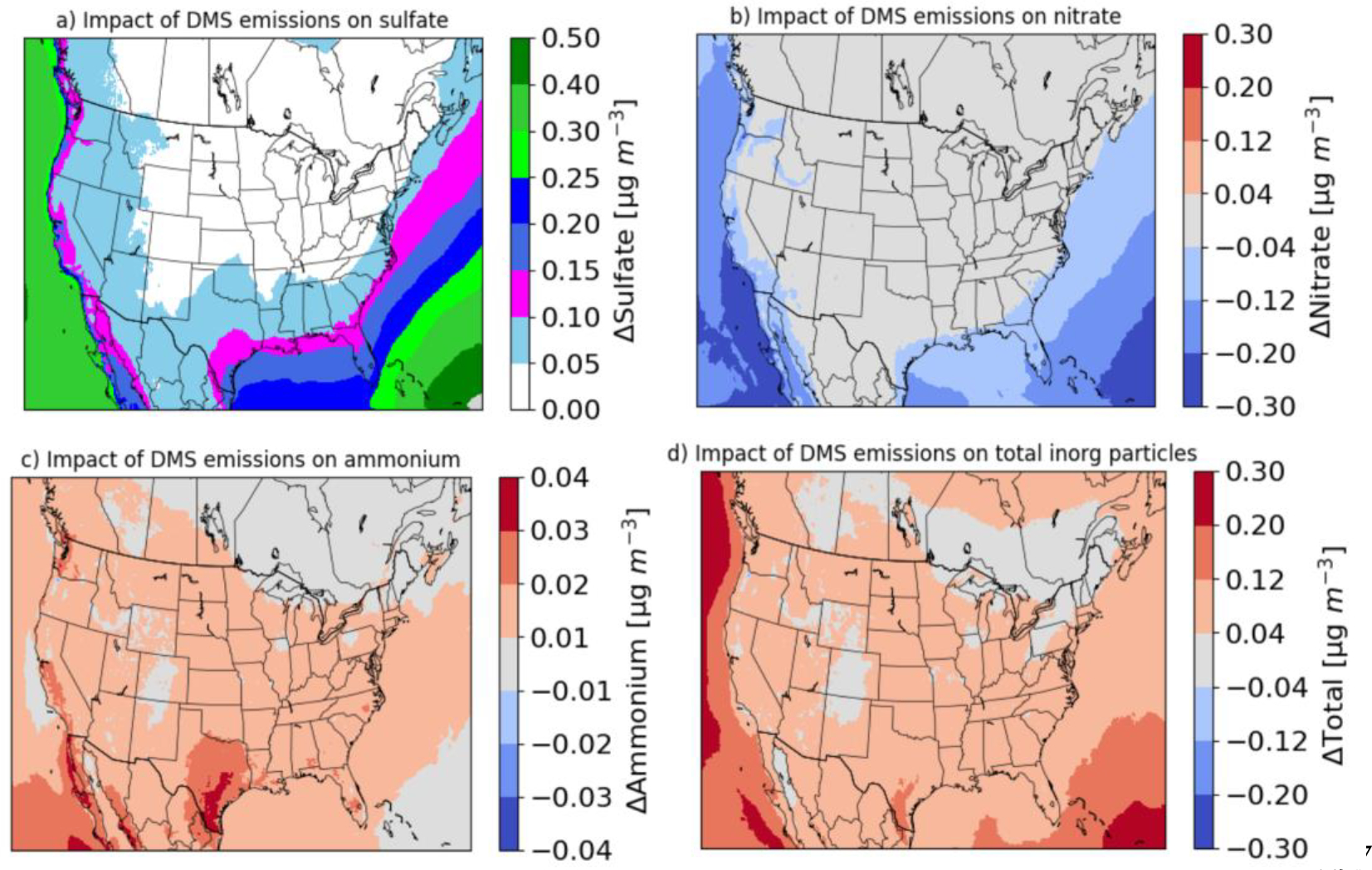
Predicted annual mean changes (WDMS – NODMS) in surface (a) sulfate, (b) nitrate, (c) ammonium, and (d) total inorganic particle concentrations by DMS emissions. Changes in inorganic particle concentrations are calculated as the net effect due to increases in sulfate and ammonium and decreases in nitrate concentrations.

Predicted annual mean changes (WDMS – NODMS) in surface sulfate, nitrate, ammonium, and total inorganic (sulfate + nitrate + ammonium) particle concentrations due to DMS emissions are shown in Figure 2. DMS emissions enhance sulfate concentrations not only over seawater but also over land area. The largest enhancements occur over seawater and then gradually decrease over the interior portion of the modeling domain. DMS is emitted from the ocean and has a relatively short lifetime (~0.5 day; [57]); thus, the highest enhancements occur over seawater. The impact is particularly high over the Pacific Ocean due in part to the prevailing wind direction carrying the impact of western boundary conditions into the modeling domain. DMS emissions enhance sulfate concentrations by 0.10–0.50 μg/m^3^ (12–67%) over a large portion of seawater, with a mean over-ocean enhancement of 0.24 μg/m^3^ (36%). DMS emissions also enhance sulfate concentrations by 0.05–0.25 μg/m^3^ over a large area of land, with a mean enhancement of 0.055 μg/m^3^ (9%). The impacts near coastal areas are greater than those over the interior portion of land. The largest impacts over land occur in coastal areas of Washington, Oregon, California, southern Texas, and Florida, in which they generally increase sulfate in some areas by more than 30–40%. They also increase sulfate by up to ~50% in some grid-cells along the coastal areas. Considering the entire state, DMS emissions increase annual mean sulfate by 0.15 μg/m^3^ (23%) over Florida, 0.11 μg/m^3^ (24%) over California, 0.09 μg/m^3^ (25%) over Oregon, and 0.09 μg/m^3^ (25%) over Washington, and 0.09 μg/m^3^ (9%) over Texas. While the impacts over some coastal states are relatively large, the impacts over many interior states covering large areas of the modeling domain are relatively small (<0.05 μg/m^3^; ~4–6%).

DMS emissions decrease nitrate concentrations by 0.04–0.28 μg/m^3^ (6–67%) over seawater and 0– 0.28 μg/m^3^ (0–69%) over coastal areas and increase ammonium concentrations by up to 0.04 μg/m^3^ (~14% at the location of the maximum increase). Overall, they decrease mean nitrate by 0.13 μg/m^3^ (39%) over the entire seawater and 0.016 μg/m^3^ (5%) over the entire land area and increase annual mean ammonium by 0.013 μg/m^3^ (35%) over the entire seawater and 0.012 μg/m^3^ (7%) over the entire land area. Ammonia preferentially reacts with sulfuric acid forming ammonium sulfate or bisulfate [9]. Any remaining ammonia then reacts with nitric acid forming ammonium nitrate. Ammonia concentrations, especially over seawater tend to be limited and are consumed by the additional sulfuric acid produced by DMS emissions increasing both the ammonium and sulfate concentrations and decreasing nitrate concentrations. Ammonium nitrate is replaced with ammonium sulfate or bisulfate; thus, the impact on ammonium tends to be smaller than the impacts on sulfate and nitrate. The impact of DMS emissions on total inorganic particles is calculated as the net effect due to increases in sulfate and ammonium and decreases in nitrate concentrations. The impacts on total inorganic particles are lower than those on sulfate due to the decreases in nitrate concentrations. However, DMS emissions still increase annual mean total inorganic particles by 0.04–0.30 μg/m^3^ (5–33%) over seawater and 0.04–0.20 μg/m^3^ (2–20%) over land.

We can compare our results to estimates from previous global and regional scale modeling studies (Table 2). Using annual results of the GEOS-Chem model, Park et al. [13] reported that natural sulfur emissions can enhance ammonium sulfate by 0.11 μg/m^3^ over the western and eastern U.S. In our study, DMS emissions increase ammonium sulfate by 0.07 μg/m^3^ over land areas of the modeling domain. Thus, the impacts in our study are lower than the values presented in Park et al. [13] due to model-to-model and emissions differences (they used natural sulfur emissions from ocean as well as other sources). Mueller et al. [18] used the CMAQv4.6 model and reported that natural emissions can enhance ammonium sulfate by 0.12 μg/m^3^ in winter and 0.27 μg/m^3^ in summer over the modeling domain. In our study, DMS emissions increase ammonium sulfate by 0.08 μg/m^3^ in winter and 0.18 μg/m^3^ in summer over the modeling domain. Thus, the impacts shown in our study are lower than those presented in Mueller et al. [18] due in part to the fact that they used natural sulfur emissions from ocean as well as other sources. Using CMAQv5.3.3 results over the Northern Hemisphere, Zhao et al. [20] reported that DMS emissions enhance annual mean sulfate by 0.08 μg/m^3^ across the entire U.S. In this study, DMS emissions increase annual mean sulfate by 0.055 μg/m^3^ over the land area of the modeling domain. The impacts are also less than the findings by Zhao et al. [20] due to differences in meteorological conditions between the simulation years, grid size, and model to model differences (two different versions of the model were used in these studies).

### 3.2 Seasonal impacts of DMS emissions on surface sulfate

**Figure 3.**
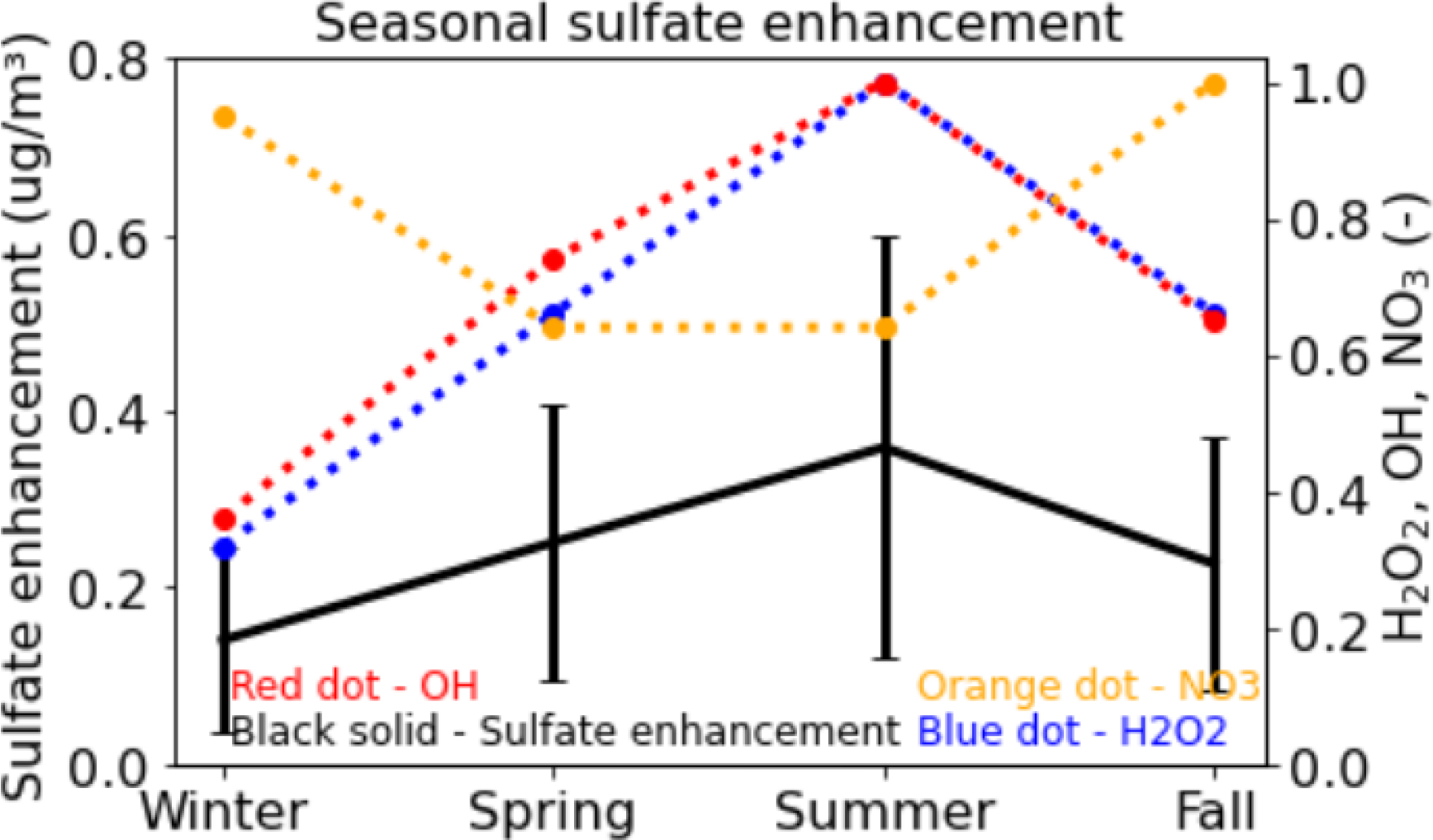
Seasonal impacts of DMS emissions on sulfate over seawater. H_2_O_2_, OH and NO_3_ concentrations shown on the right y-axis are dimensionless. Normalized values are calculated by dividing the actual concentrations by their maximum concentrations and are shown on y-axis. Winter represents December-February, spring represents March-May, summer represents June-August, and fall represents September-November. Solid vertical lines represent standard deviation of sulfate enhancement.

Seasonal mean impacts of DMS emissions on sulfate along with OH, NO_3_ and H_2_O_2_ concentrations over seawater are shown in Figure 3. DMS enhances sulfate in each season with the greatest enhancement occuring in summer and the smallest in winter. The enhancement in summer is ~2.5 times greater than that in winter, which is consistent with increased emissions and increased oxidation. The formation of sulfate from DMS occurs in two steps. In the first step, DMS is oxidized into SO_2_ via the OH, NO_3_ and Cl initiated reactions. DMS oxidation via the Cl initiated reaction is generally smaller than the other pathways since Cl levels are lower than other oxidants. In the second step, the resulting SO_2_ is oxidized into sulfate via the gas-phase reaction with OH and aqueous-phase reactions with H_2_O_2_ and other oxidants. Sulfur oxidation by H_2_O_2_ in cloud and OH in gas-phase are important processes for producing sulfate in the atmosphere [9]. While NO_3_ levels are higher in winter and fall, both OH and H_2_O_2_ concentrations peak in summer. Thus, both the DMS and SO_2_ oxidation can occur efficiently producing the highest sulfate enhancement in summer. In contrast, both OH and H_2_O_2_ concentrations are the smallest in winter producing the lowest enhancement of sulfate.

**Figure 4.**
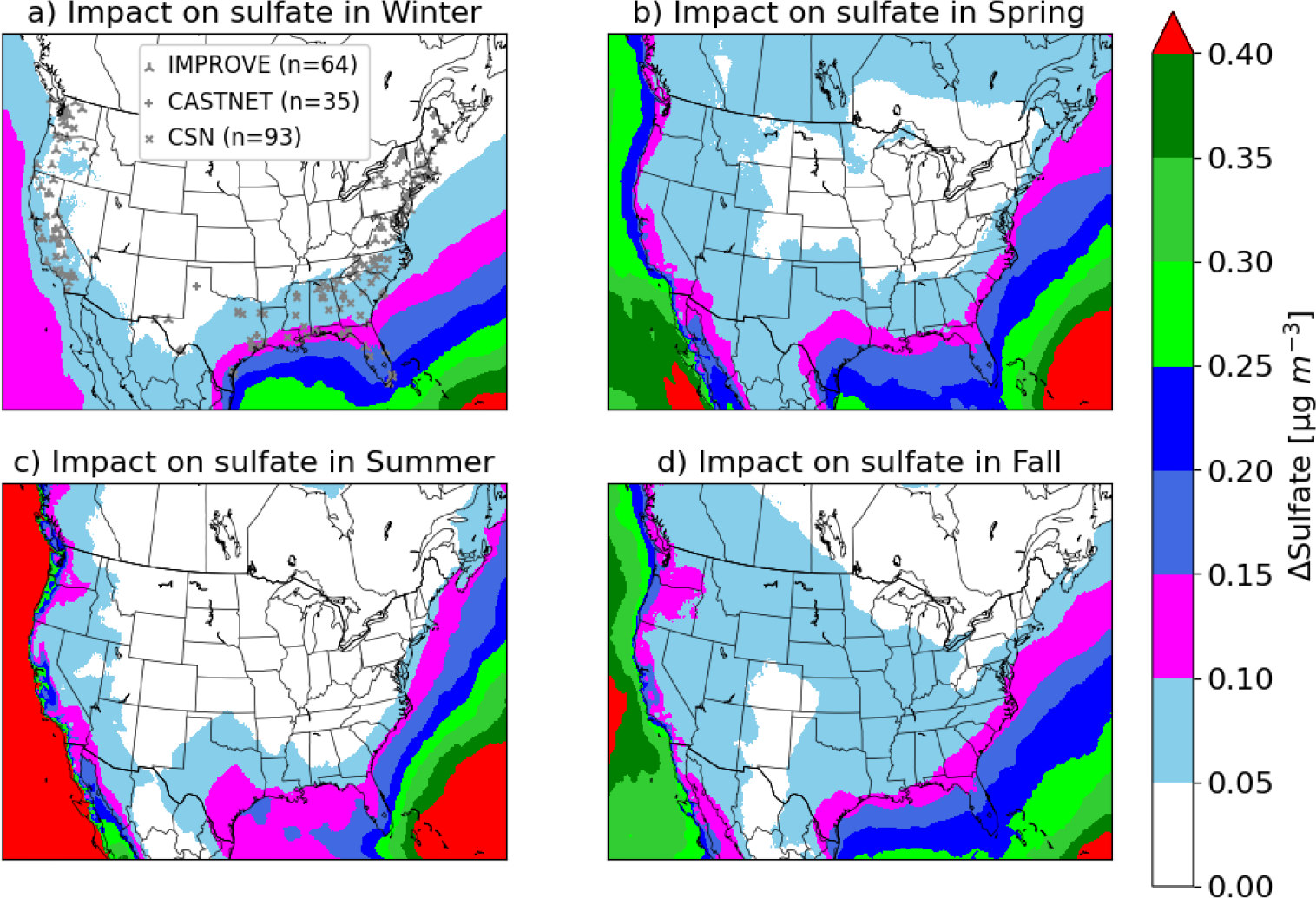
Spatial distribution of the seasonal impacts of DMS emissions on sulfate in (a) winter, (b) spring, (c) summer, and (d) fall. Figure 4(a) shows monitoring locations for observed data used in Figure 8.

The spatial distribution of the seasonal impacts is shown in Figure 4. Consistent with the annual results shown in Figure 2a, larger impacts occur over seawater than those over land in all seasons. DMS induced sulfate enhancements are lower in winter over most areas than those in other seasons. In winter, however, DMS emissions increase sulfate over the Gulf of Mexico and part of the Atlantic Ocean by larger margins than those over the Pacific Ocean – leading to higher sulfate concentrations in the winter. In other seasons, DMS increases sulfate over the Pacific Ocean by larger margins due to the contribution from the western boundary condition, higher DMS emissions, and higher oxidant levels. The impacts of DMS emissions over seawater are the highest in summer; however, the impacts tend to be localized over coastal areas. The spatial distributions of the impacts on sulfate are different in spring and fall than those in summer. The mean enhancements over seawater in spring and fall are lower than those in summer. However, DMS emissions produce greater impacts over a large interior portion of land in spring and fall due to higher wind speed, which can effectively transport the resulting sulfate to land. Wind speed is the highest in winter; however, sulfate enhancements are the lowest; thus, the impacts over land are not as widespread as predicted in spring and fall. The average DMS induced sulfate enhancements over land are 0.04 μg/m^3^ (6%), 0.07 μg/m^3^ (8%), 0.05 μg/m^3^ (11%), and 0.06 μg/m^3^ (13%) in winter, spring, summer, and fall season, respectively.

### 3.3 Diurnal variation of the sulfate enhancement

**Figure 5.**
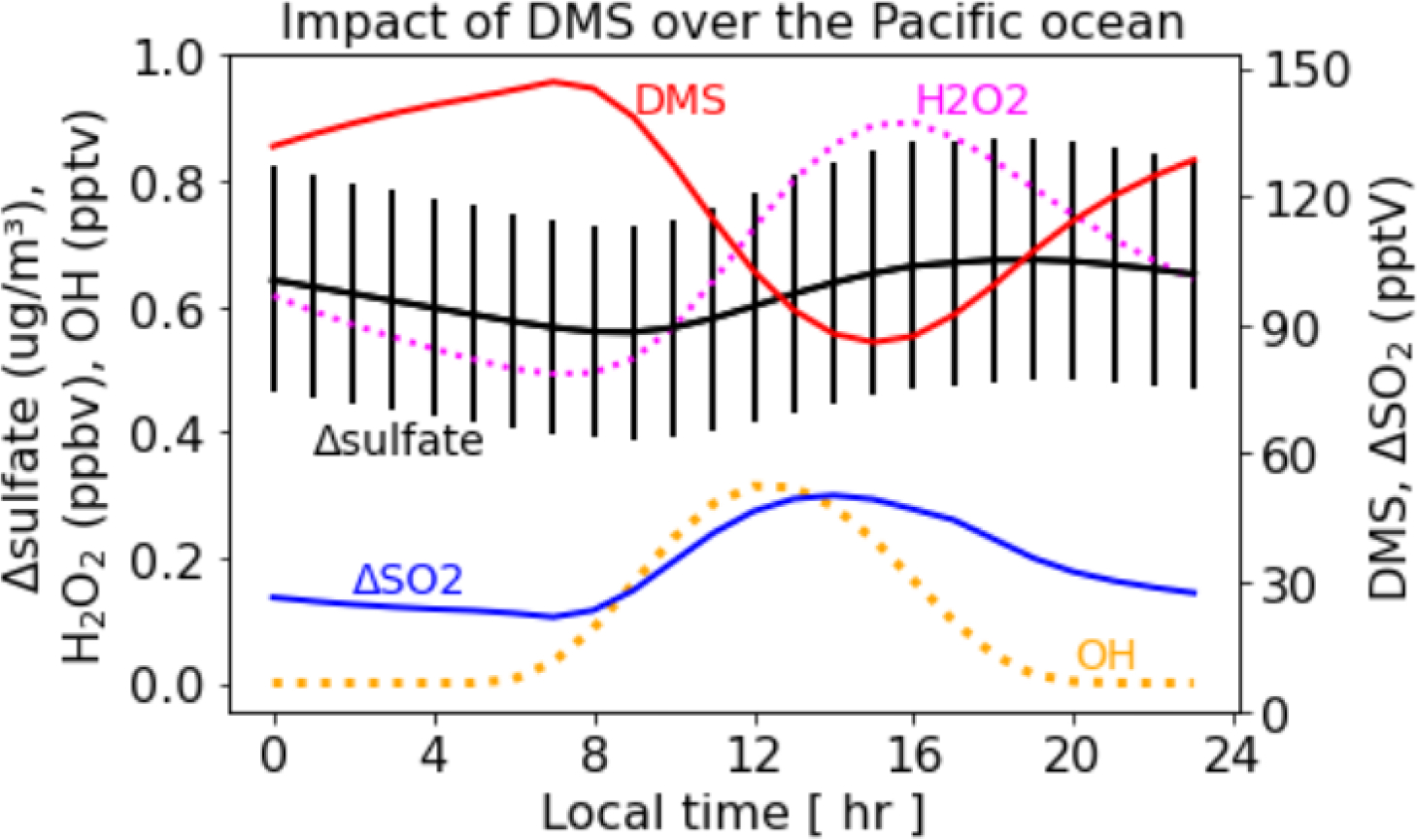
Diurnal variation of the impacts of DMS emissions on sulfate over the Pacific Ocean in July. All grid-cells over the Pacific Ocean (including the Gulf of California) are used for the calculation. Solid vertical lines represent standard deviation of sulfate enhancement.

To examine the diurnal variation of DMS induced sulfate, we select all grid-cells over the Pacific Ocean (including the Gulf of California) in July. Diurnal variation of H_2_O_2_, OH, DMS, and SO_2_ and sulfate produced by DMS emissions over the Pacific Ocean is shown in Figure 5. OH concentrations remain low at night, start increasing during the day, and peak around noon. H_2_O_2_ concentrations are also lower at night, increase during the day, and peak during mid-afternoon. DMS concentrations slowly increase from mid-night to morning as DMS is continuously emitted but only being oxidized by NO_3_ at night, which is lower in summer than in winter. The DMS concentrations decrease sharply during the day, reach their lowest level around mid-afternoon, and then increase again. The decrease in DMS concentrations during the day occurs due to higher OH concentrations. SO_2_ levels remain relatively flat during the night despite SO_2_ production from the oxidation of DMS by NO_3_. SO_2_ concentrations start increasing during the day as more DMS is oxidized by higher daytime OH levels, peak at mid-afternoon, then decrease at night. Sulfate from the DMS emissions tends to decrease slowly during the night as little sulfate is produced since OH and H_2_O_2_ are lower at night. Sulfate levels increase during the day and peak in late afternoon due to increased oxidation by OH (in gas-phase) and H_2_O_2_ in cloud. Thus, DMS induced sulfate tends to be higher during the day and lower at night.

### 3.4 Impact of DMS emissions on sulfate aloft

**Figure 6.**
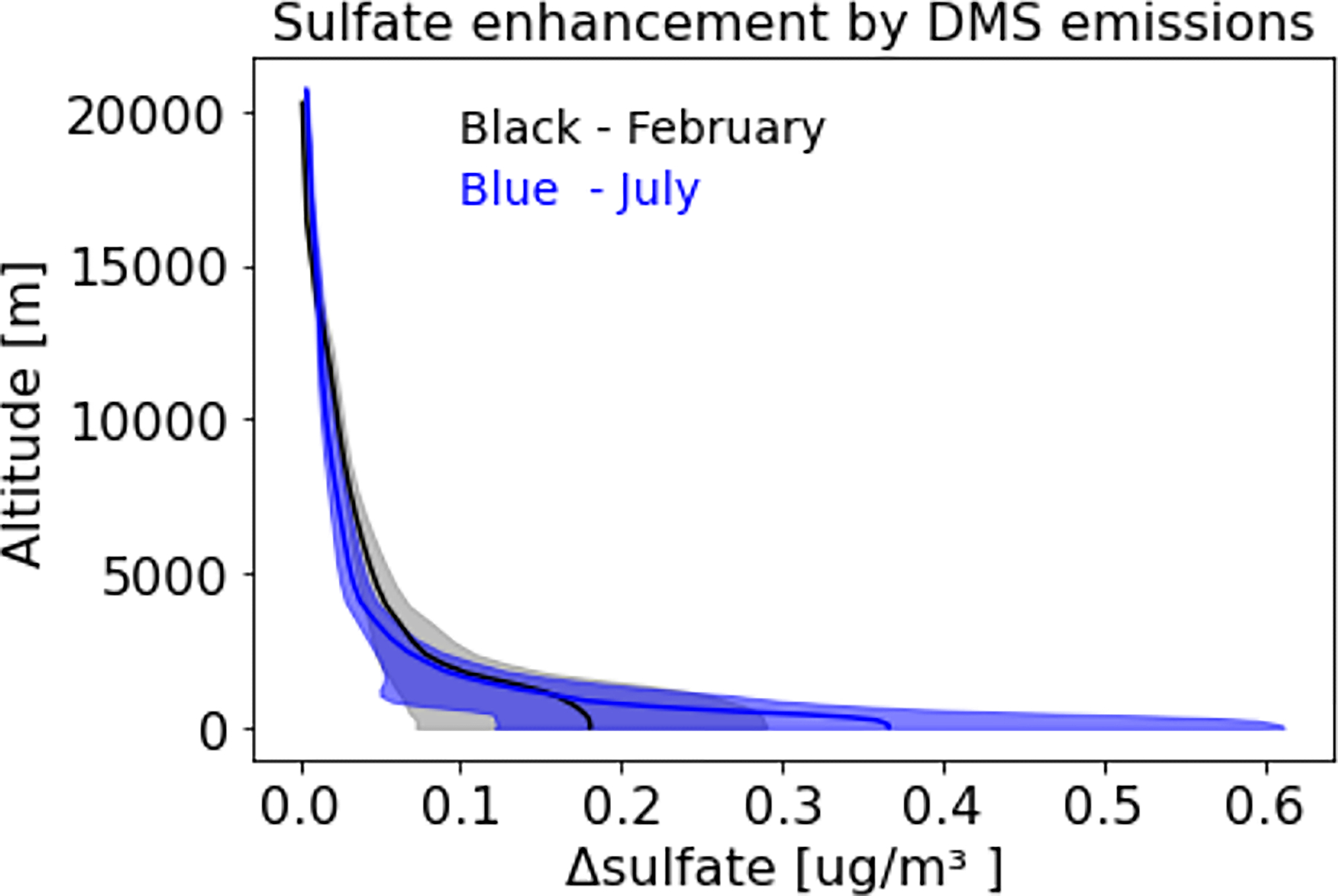
Impact of DMS emissions on sulfate aloft. Solid lines represent mean and shaded areas represent standard deviation of sulfate enhancement.

To examine the impact of DMS emissions aloft, we focus on a winter (February) and a summer (July) month. DMS induced sulfate enhancements over the entire oceanic area with altitude are shown in Figure 6. DMS emissions enhance surface layer sulfate by ~0.2 μg/m^3^ in February and ~0.4 μg/m^3^ in July. These emissions consistently produce more sulfate in summer than in winter. DMS induced sulfate enhancements are highest at the surface and decrease with altitude, diminishing to ~10–20% at an altitude of ~5 km. Thus, the DMS emissions enhance sulfate not only at the surface layer but also aloft, though its impact tends to be limited in the lower troposphere.

### 3.5 Impact of DMS emissions and boundary conditions on sulfate

**Figure 7.**
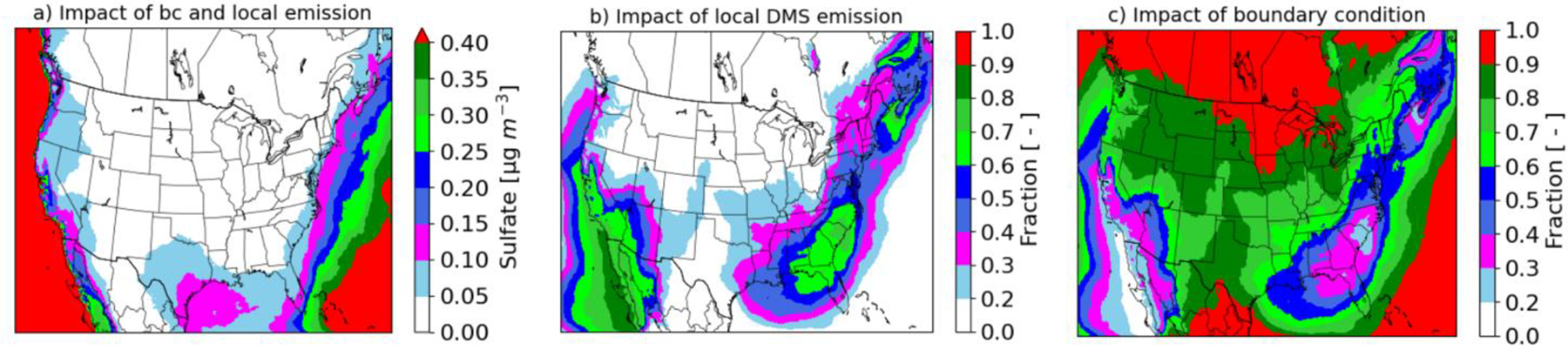
(a) Combined impact of DMS emissions and boundary conditions on sulfate (b) fractional impact of within-domain DMS emissions on sulfate, and (c) fractional impact of outside-domain DMS emissions (specified through boundary conditions) on sulfate in July..

To understand the relative importance of in-domain emissions and transport on sulfate, we complete an additional sensitivity simulation similar to WDMS, but without DMS emissions in the 12 km domain. The additional sensitivity simulation, therefore, only has DMS and products from the boundary conditions, so we refer to it as BCDMS. The in-domain contribution is calculated as the difference between WDMS (both BC and local emissions) and BCDMS (only BC). The boundary contribution is calculated as the difference between BCDMS (only BC) and NODMS (no DMS). The combined impacts of in-domain DMS emissions and boundary conditions (WDMS - NODMS) on July mean sulfate are shown in Figure 7a. Consistent with Figures 2a and 4c, DMS emissions and boundary conditions produce higher impacts over seawater than over land, and they also enhance sulfate in many coastal areas. Figures 7b and 7c show the fractional contributions of in-domain DMS emissions (WDMS – BCDMS) and outside-domain DMS emissions (BCDMS – NODMS) to the total sulfate enhancement (WDMS – NODMS) shown in Figure 7a. In-domain DMS emissions increase sulfate over seawater by fractions of >0.6 over the southern Pacific coast, the southern Atlantic coast, and the coast of Maine (Figure 7b). In contrast, boundary conditions enhance sulfate over seawater by fractions of >0.6 over the northern Pacific coast, further offshore in the Atlantic/Gulf of Mexico, the tip of Florida, and over a large land area (Figure 7c). While the combined impacts of DMS emissions and boundary conditions over the interior portion of the modeling domain are relatively small (< 0.05 μg/m^3^), the impact over this region is dominated by boundary conditions, i.e. the long-range transport of sulfate formed from DMS emissions over the hemisphere. These results suggest that boundary conditions play an important role on sulfate enhancements not only near the boundaries but also over the interior portion of the modeling domain. Thus, it is important to employ appropriate boundary conditions in the model; otherwise, regional-scale model predictions will not reflect the full effects of DMS emissions on the relevant temporal and spatial scales. It should be noted that boundary conditions obtained with the DMS emissions from the hemispheric model includes the impacts on DMS, SO_2_ and sulfate. These results suggest that enhancements of sulfate from DMS on the Pacific Northwest of 0.25–0.3 μg/m^3^ are primarily from emissions outside the domain. This is consistent with large fluxes between the Pacific Northwest and Alaska from May through August as evident in Zhao et al. [20] and Hulswar et al. [58].

### 3.6 Comparison with observed sulfate

The largest impact of DMS emissions on land occurs in coastal areas. Thus, we focus on model performance in coastal states and compare model predictions with observed data in four separate groups: (1) the Pacific-coast states (Washington, Oregon, California), (2) the Gulf-coast states (Texas, Louisiana, Mississippi, Alabama), (3) Florida, and (4) the Atlantic-coast states (Georgia, South Carolina, North Carolina, Virginia, Maryland, Delaware, New Jersey, New York, Connecticut, Rhode Island, Massachusetts, New Hampshire, Maine). We calculate monthly mean observed sulfate concentrations by combining data from IMPROVE (Interagency Monitoring of Protected Visual Environments), CASTNET (Clean Air Status and Trends Network) and CSN (Chemical Speciation Network) sites within each group. We then calculate mean sulfate concentrations without and with DMS emissions by combining predictions at all sites within the group and compare them to the corresponding observed data.

**Figure 8.**
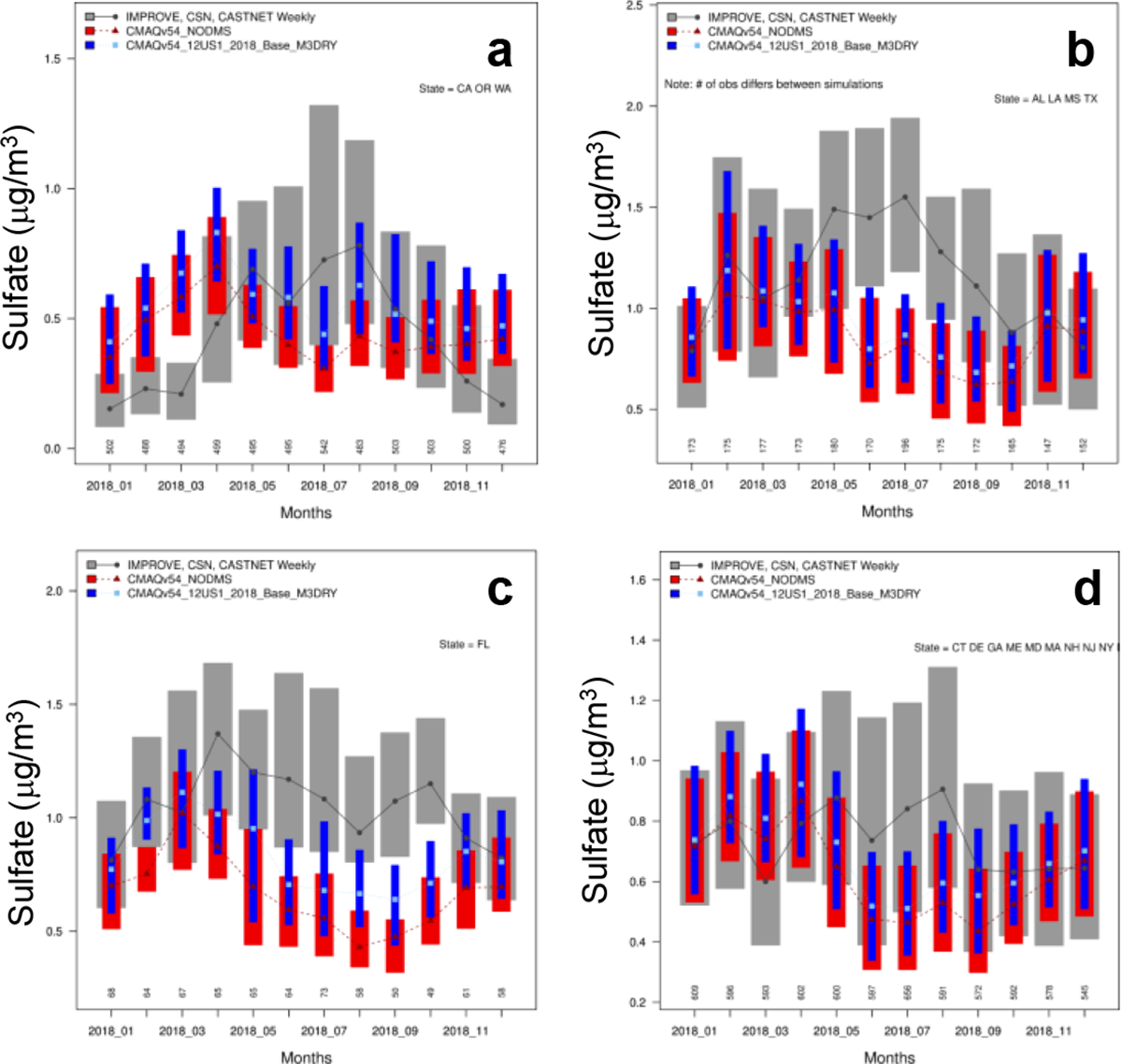
A comparison of predicted sulfate without and with DMS emissions to observed sulfate in (a) the Pacific-coast states, (b) the Gulf-coast states, (c) Florida, and (d) the Atlantic-coast states. Boxplot of observed sulfate (grey boxes and lines) from IMPROVE, CSN and CASTNET; model without DMS emissions (red boxes and lines); and model with DMS emissions (blue boxes and lines) by month. The shaded box represents the interquartile range (25% to 75%) of the data and the point indicates the median value. The numbers below each box indicate the total number of observations available for that month from the three networks. See Figure 4(a) for the locations of monitoring sites.

Figure 8 shows that the effect on performance varies by region and season. In all regions, the NODMS underpredicts May through October observations. Adding DMS improves the model performance. For the cooler months, the performance is mixed because the base model overpredicts in some places and underpredicts in others. For cooler months (January-April, November, December), NODMS overpredicts compared to observations so adding DMS deteriorates the model performance. In the Gulf-coast states, NODMS only overpredicts in January and December and DMS emissions improve the comparison with observed data in all other months. A similar pattern is also noticeable in Florida and the Atlantic-coast states. Thus, additional sulfate from DMS emissions tends to deteriorate the model performance in cooler months while improving it in warmer months.

**Figure 9.**
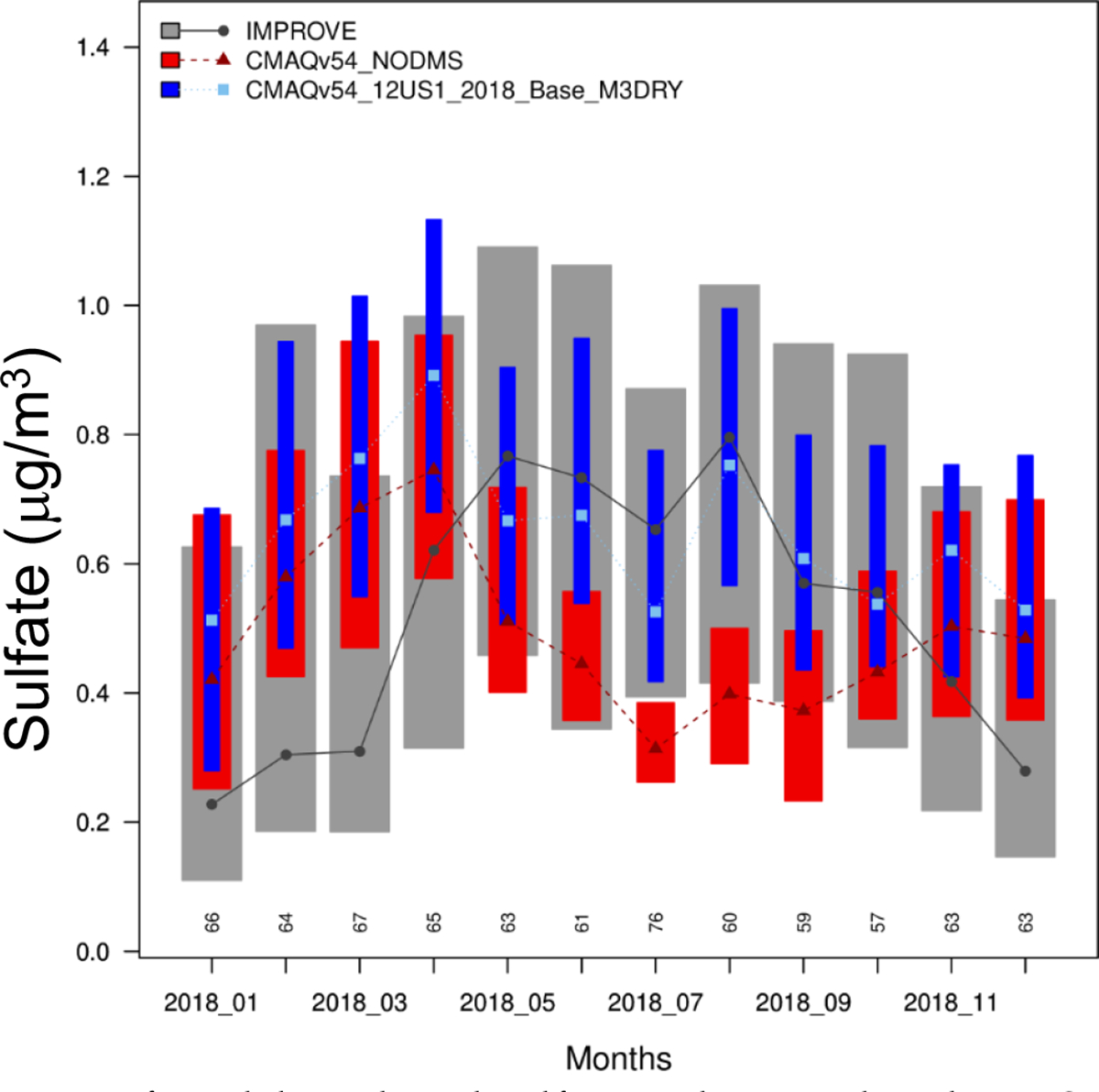
A comparison of model predicted sulfate without and with DMS emissions to observed sulfate at 7 coastal sites (IMPROVE sites located within 10-km of the coast) in the California (PORE1, REDW1), Washington (MAKA2, OLYM1, PUSO1), and Florida (CHAS1, SAMA1). Boxplot of observed sulfate (grey boxes and lines); model without DMS emissions (red boxes and lines); and model with DMS emissions (blue boxes and lines) by month. The shaded box represents the interquartile range (25% to 75%) of the data and the point indicates the median value. The numbers below each box indicate the total number of observations available for that month.

To further examine the impact on model performance, we isolate IMPROVE sites located within 10-km from the coastline in the Pacific-coast states and Florida and compare monthly mean sulfate concentrations without and with DMS emissions to observed data (Figure 9). Similar to Figure 8, DMS emissions deteriorate the model performance in cooler months. However, they improve the comparison in warmer months by larger margins than those shown in Figure 8. Thus, DMS emissions can improve the model performance by a larger margin near the coastal areas than the interior areas.

### 3.7 Changes in DMS initiated sulfate enhancement with horizontal grid resolution

**Figure 10:**
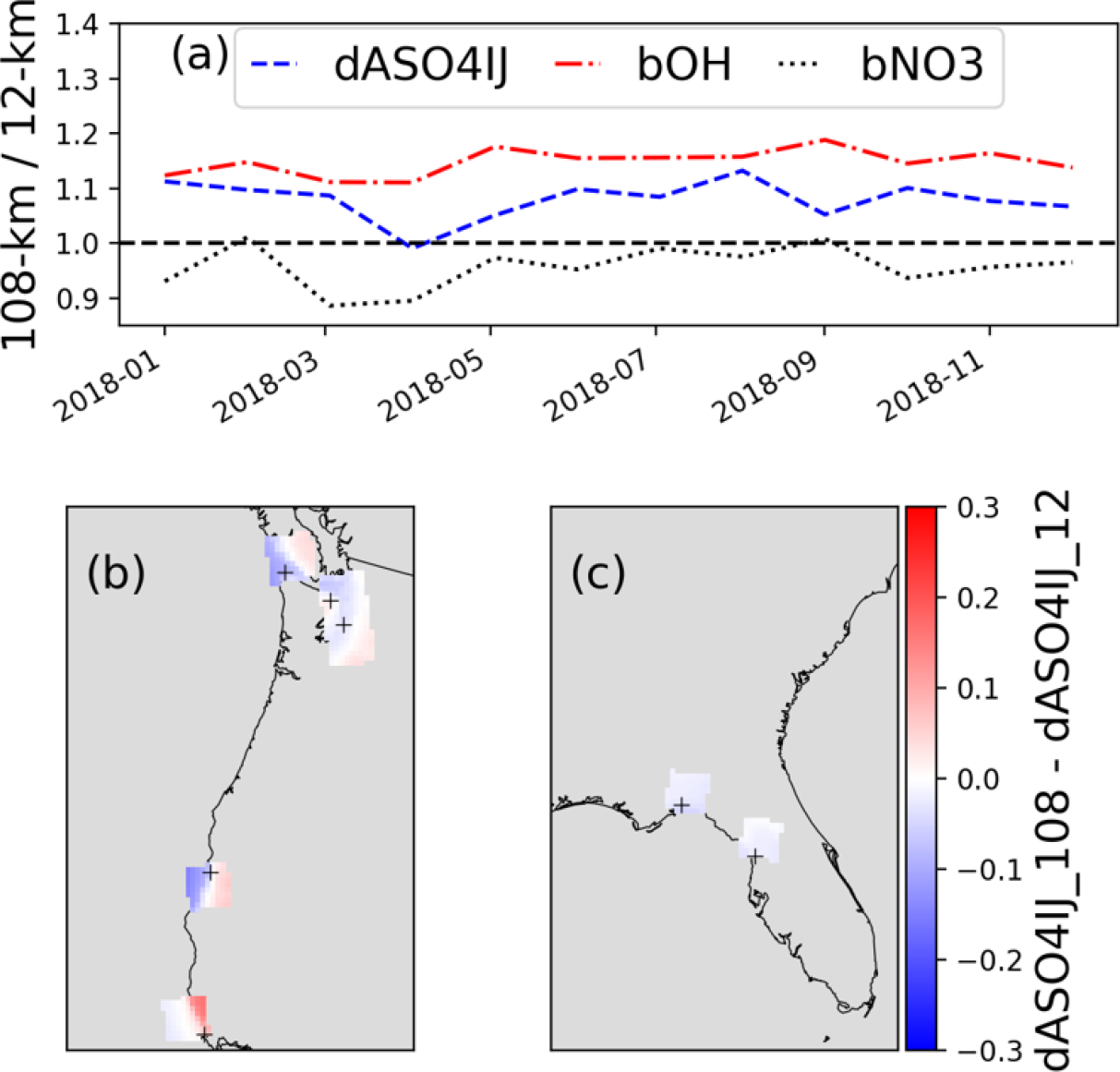
(a) Ratios of 108km to 12km sulfate enhancement (dSO4, blue solid), DMS simulation OH concentration (bOH, red dashed), and DMS simulation NO_3_ (bNO_3_, blue dotted) for ocean cells within the 12US1 domain. dSO4, bOH, and bNO_3_ are shown on the y-axis and dates are shown on the x-axis (b-c) annual mean difference of sulfate enhancements between 108-km and 12-km horizontal grid resolutions zoomed in on the Pacific coast (b) and Florida (c). Seven IMPROVE sites were used in the calculation: PORE1 and REDW1 in California, MAKA2, OLYM1, PUSO1 in Washington, and CHAS1, SAMA1 in Florida.

Most of the prior studies employed large scale models using coarse horizontal grid resolution to examine the impact of oceanic DMS emissions on sulfate. In this study, we use a finer horizontal grid resolution (12-km) over the continental U.S. We also conducted CMAQ simulations without and with DMS emissions over the Northern hemisphere using coarse (108-km) horizontal grids from which we created boundary conditions for model simulations over the continental U.S. Here, we examine the impacts of DMS emissions on sulfate using results from simulations obtained with 12-km and 108-km horizontal grids. We calculate DMS initiated monthly mean sulfate enhancements over the entire seawater of the modeling domain covering the continental U.S. using both the 108-km and 12-km horizontal grids. Over the entire seawater, ratios of sulfate enhancements with 108-km grids to 12-km horizontal grids are greater than 1.0 (Figure 10a) which indicates that model with 108-km grids produces slightly more DMS initiated sulfate than the model with 12-km grids. Ratios of model OH and NO_3_ concentrations over the seawater with 108-km and 12-km grids are also shown in Figure 10a. The OH concentrations over the entire seawater are consistently greater with 108-km grids than those with 12-km grids (ratio >1.0). In contrast, the NO_3_ concentrations over the entire seawater are generally lower with 108-km grids than those with 12-km grids (ratio <1.0). The grid resolution affects many parameters in the model which in turn affect DMS initiated sulfate enhancements. However, OH appears to play an important role in affecting the DMS initiated sulfate enhancement. The higher DMS initiated sulfate with 108-km grids occurs primarily due to additional DMS oxidation via higher OH concentrations and additional SO_2_ oxidation with OH concentrations. However, such enhancements with 108-km grids do not occur in all areas. For example, the model with 108-grids generally produces more sulfate enhancements over the Pacific Ocean. In contrast, the model with 108-km grids produces less sulfate enhancements over portions of the Gulf of Mexico and the Atlantic Ocean primarily due to reduced DMS oxidation by lower NO_3_ concentrations and reduced SO_2_ oxidation by lower H_2_O_2_ concentrations. Thus, DMS initiated sulfate enhancements are dependent on grid resolution and geographic location. To further illustrate the complexity of evaluating the impacts of grid-resolution at individual sites, the annual mean difference of DMS initiated sulfate at the seven coastal IMPROVE sites (same sites used in Figure 9) are shown in Figure 10b–c. Each 108-km grid-cell contains 81 approximately 12-km grid-cells. The model with 108-km grids produces lower sulfate enhancements consistently at the two sites in Florida than those with the model with 12-km grids. However, the impacts at the western U.S. sites are mixed within 108-km grid-cells. The model with 108-km grids produces more sulfate enhancements at some sites while producing less sulfate enhancements at other sites. Such results show the importance of considering grid-resolution in evaluating the impacts of DMS initiated sulfate enhancements.

## 4. Conclusions

We examined the impacts of DMS emissions on sulfate concentrations over the continental U.S. and surrounding areas by using CMAQv5.4 with a horizontal grid resolution of 12-km and boundary conditions derived from a comparable hemispheric-scale model. DMS emissions increase annual mean sulfate concentrations over seawater and land areas, with the largest impact over seawater. In-domain DMS emissions increase sulfate more over coastal areas than over interior portions of land, with the largest impacts occuring over Washington, Oregon, California, and Florida. Seasonally, the largest enhancements over seawater occur in summer and the lowest in winter. These enhancements help reduce the low bias in modeled sulfate at coastal sites in California, Washington, and Florida. The over seawater enhancements in spring and fall are lower than those in summer but greater than those in winter. However, enhanced sulfate can occur deeper into land areas in spring and fall due to the prevailing higher wind speeds, causing slightly higher enhancements than those in summer. DMS emissions increase sulfate throughout the lower troposphere, though the impacts at higher altitudes decrease sharply compared to those near the surface. Predicted sulfate with DMS deteriorates the model performance in cooler months while improving it in warmer months. DMS plays a larger role in affecting the model performance in coastal areas. Sulfate enhancements from DMS emissions on a hemispheric scale represented through boundary conditions for the 12 km domain play an important role in affecting sulfate enhancements, not only near the boundaries, but also in the interior portion of the modeling domain.

As indicated by our results, the oxidation of DMS in the troposphere and subsequent conversion to SO_2_ are key processes for the formation and growth in sulfur-containing aerosols in the marine boundary layer which then can also undergo long-range transport and influence background aerosol sulfate levels in continental areas. Though our current implementation of DMS chemistry captures the dominant oxidation pathways via OH and NO_3_, future enhancements to the representation of halogen chemistry in the model enable more complete representation of DMS oxidation by reactive halogens as well as multi-phase pathways [16]. Additionally, Veres et al. [59] recently identified hydroperoxymethyl thioformate (HPMTF) as a new DMS oxidation product in measurements performed during the Atmospheric Tomography mission over marine environments. The addition of HPMTF and its oxidation pathways could potentially slow down DMS oxidation to SO_2_ and thus reduce the abundance of SO_2_ and sulfate in areas of DMS emissions but enhance their levels in downwind cloud-free regions where HPTMF can continue to oxidize to produce SO_2_ and sulfate. In contrast, Novak et al. [60] suggest that in the cloudy-marine boundary layer, the HPMTF lifetime is considerably reduced due to uptake in cloud droplets, limiting the production of SO_2_. However, paucity of direct observations of the full suite of DMS oxidation products across the variable conditions of the marine boundary layer make it challenging to adequately constrain the reaction pathways and SO_2_ yields from DMS oxidation. Future efforts will focus on enhancing the representation of these pathways, as better estimates of these multiphase chemical pathways evolve.

## Figures and Tables

**Table 1.** Gas-phase chemical reactions for DMS oxidation in CMAQ

No.	Reaction	Rate Expression (cm^3^ molecule^−1^ sec^−1^)	References
1	DMS + OH = SO_2_ + .. (abstraction channel)	k = 1.12 × 10^−11^ e^−250/T^ T = temperature in Kelvin	[53]
2	DMS + OH = 0.75 × SO_2_ + .. (addition channel)	k_o_ = 1.99 × 10^−39^ e^−5270/T^ k_∞_ = 1.26 × 10^−10^ e^+340/T^ k={k_o_[M]/(1+k_o_[M]/k_∞_)} F^z^ Z={(1/N)+log_10_[k_o_ [M]/k_∞_]^2^}^−1^ F = 1.0 and N = 1.0 [M] = total pressure, molecules/cm^3^	[53]
3	DMS + NO_3_ = SO_2_ + ..	k = 1.93 × 10^−13^ e^+520/T^	[53]
4	DMS + Cl = 0.86 × SO_2_ + ..	k = 3.4 × 10^−13^ e^+2081/T^	[54–55]

**Table 2.** A comparison of sulfate enhancement in different studies

Studies	Enhancements μg/m^3^	Sources considered	Geographic region	Season
Park et al. [13]	Ammonium sulfate 0.11	DMS from seawater, volcanoes, and biomass burning activities	Eastern and western US	Annual
This study	Ammonium sulfate 0.07	DMS from seawater	Entire land area of the modeling domain	Annual
Mueller et al. [18]	Ammonium sulfate 0.12	DMS and hydrogen sulfide from seawater, coastal wetlands, freshwater, Great Salt Lake, soils, volcanoes and fumaroles	Entire modeling domain	Winter (December-February)
This study	Ammonium sulfate 0.08	DMS from seawater	Entire modeling domain	Winter (December-February)
Mueller et al. [18]	Ammonium sulfate 0.27	DMS and hydrogen sulfide from seawater, coastal wetlands, freshwater, Great Salt Lake, soils, volcanoes and fumaroles	Entire modeling domain	Summer (June-August)
This study	Ammonium sulfate 0.18	DMS from seawater	Entire modeling domain	Summer (June-August)
Zhao et al. [20]	Sulfate 0.08	DMS from seawater	Entire US	Annual
This study	Sulfate 0.055	DMS from seawater	Entire US	Annual

## Data Availability

Data will be made available on request.
